# Auditory and vestibular function in mitochondrial patients harbouring the m.3243A>G variant

**DOI:** 10.1093/braincomms/fcae361

**Published:** 2024-10-14

**Authors:** Renae J Stefanetti, Jane Newman, Alasdair P Blain, Donella Chisari, Gráinne S Gorman, Gary Rance

**Affiliations:** Faculty of Medical Sciences, Wellcome Centre for Mitochondrial Research, Translational and Clinical Research Institute, Newcastle University, Newcastle upon Tyne, NE2 4HH, UK; National Institute for Health and Care Research Newcastle Biomedical Research Centre, The Medical School, Newcastle upon Tyne, NE2 4HH, UK; NHS Highly Specialised Service for Rare Mitochondrial Disorders of Adults and Children, Newcastle upon Tyne Hospitals NHS Foundation Trust, Newcastle upon Tyne, NE2 4HH, UK; Faculty of Medical Sciences, Wellcome Centre for Mitochondrial Research, Translational and Clinical Research Institute, Newcastle University, Newcastle upon Tyne, NE2 4HH, UK; National Institute for Health and Care Research Newcastle Biomedical Research Centre, The Medical School, Newcastle upon Tyne, NE2 4HH, UK; NHS Highly Specialised Service for Rare Mitochondrial Disorders of Adults and Children, Newcastle upon Tyne Hospitals NHS Foundation Trust, Newcastle upon Tyne, NE2 4HH, UK; Faculty of Medical Sciences, Wellcome Centre for Mitochondrial Research, Translational and Clinical Research Institute, Newcastle University, Newcastle upon Tyne, NE2 4HH, UK; National Institute for Health and Care Research Newcastle Biomedical Research Centre, The Medical School, Newcastle upon Tyne, NE2 4HH, UK; Department of Audiology & Speech Pathology, Faculty of Medicine, Dentistry and Health Sciences, The University of Melbourne, Parkville, VIC 3010, Australia; Faculty of Medical Sciences, Wellcome Centre for Mitochondrial Research, Translational and Clinical Research Institute, Newcastle University, Newcastle upon Tyne, NE2 4HH, UK; National Institute for Health and Care Research Newcastle Biomedical Research Centre, The Medical School, Newcastle upon Tyne, NE2 4HH, UK; NHS Highly Specialised Service for Rare Mitochondrial Disorders of Adults and Children, Newcastle upon Tyne Hospitals NHS Foundation Trust, Newcastle upon Tyne, NE2 4HH, UK; Department of Audiology & Speech Pathology, Faculty of Medicine, Dentistry and Health Sciences, The University of Melbourne, Parkville, VIC 3010, Australia

**Keywords:** auditory neuropathy, hearing impairment, m.3243A>G mitochondrial disease, speech perception, vestibular dysfunction

## Abstract

Hearing impairment is a frequent clinical feature in patients with mitochondrial disease harbouring the pathogenic variant, m.3243A>G. However, auditory neural dysfunction, its perceptual consequences and implications for patient management are not established. Similarly, the association with vestibular impairment has not yet been explored. This case–control study investigated in 12 adults with genetically confirmed m.3243A>G adults [9 females; 45.5 ± 16.3 years (range 18–66); 47.1 ± 21.5 hearing level, dB] compared with 12 age, sex and hearing level-matched controls with sensory (cochlear level) hearing loss [9 females; 46.6 ± 11.8 years (range 23–59); 47.7 ± 25.4 hearing level, dB]. Participants underwent a battery of electroacoustic, electrophysiologic and perceptual tests, which included pure tone audiometry, otoacoustic emissions, auditory brainstem responses, auditory temporal processing measures, monaural/binaural speech perception, balance and vestibular testing and self-reported questionnaires (dizziness and hearing disability). Our findings showed evidence of auditory neural abnormality and perceptual deficits greater than expected for cochlear pathology. Compared with matched controls with sensory hearing loss, adults with mitochondrial disease harbouring m.3243A>G had abnormal electrophysiologic responses from the VIII nerve and auditory brainstem (*P* = 0.005), an impaired capacity to encode rapidly occurring acoustic signal changes (*P* = 0.005), a reduced ability to localize sound sources (*P* = 0.028) and impaired speech perception in background noise (*P* = 0.008). Additionally, vestibular dysfunction (*P* = 0.011), greater perceived dizziness (*P* = 0.001) and reduced stance time (balance, *P* = 0.009) were also seen in participants with m.3243A>G mitochondrial disease when compared with matched counterparts. This pilot study revealed that auditory evaluation including evoked potential responses from the auditory nerve/brainstem and speech perception in noise tests should form an important part of the management for individuals with m.3243A>G-related mitochondrial disease. Those presenting with hearing impairment and symptoms concerning balance and dizziness should undergo vestibular testing and appropriate management.

## Teaser

Stefanetti *et al.* report that individuals with genetically confirmed m.3243A>G mitochondrial DNA disease commonly present with auditory dysfunction affecting both the cochlea and auditory neural systems. As such, they suffer severe speech understanding and communication difficulties in everyday life. Additionally, affected patients have vestibular dysfunction, which results in dizziness and imbalance.

## Graphical Abstract

**Graphical Abstract:**
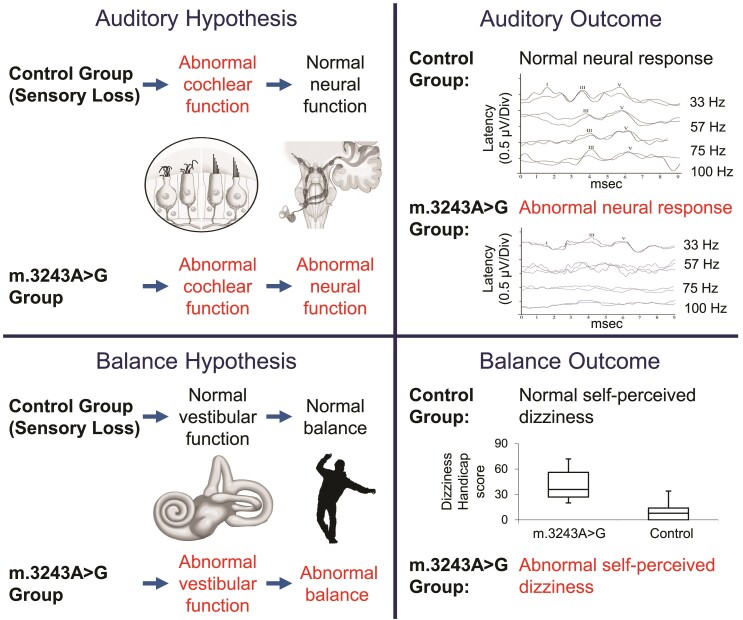

## Introduction

Mitochondrial diseases are a heterogeneous group of genetic disorders characterized by disruptions in oxidative phosphorylation and caused by pathogenic variants in both nuclear DNA–encoded and mitochondrial DNA (mtDNA)–encoded genes.^1^ Mitochondrial diseases are one of the most common neurogenetic diseases and are among the most common forms of inherited neurological disorder with an incidence of ∼1 in 4300.^2^

A common manifestation of mitochondrial disease is hearing impairment, which can occur in both isolation (non-syndromic) and as a feature of systemic disease (syndromic).^3^ While not all mtDNA genetic defects cause impaired sound detection thresholds,^4^ diverse genetic aetiologies are responsible for mitochondrial-related hearing impairment, with the most common being the m.3243A>G pathogenic mtDNA variant within *MT–TL1* gene, associated with extreme clinical heterogeneity including deafness, diabetes, ataxia, cardiomyopathy and myopathy.^5^ With respect to hearing loss, the m.3243A>G pathogenic variant has previously been shown to cause progressive, bilateral, symmetrical sensory hearing loss, with the level of heteroplasmy measured in muscle tissue found to correlate with the severity of hearing loss.^4^ The pathology of mitochondrial disease–related hearing loss in this case has predominantly been attributable to cochlear abnormality,^4,6,7^ resulting from dysfunction of the stria vascularis and outer hair cells (highly metabolically active cochlear structures, vulnerable to mitochondrial dysfunction).

Pathological changes in other parts of the auditory pathway have, however, been suggested in patients with mitochondrial disease. The term ‘auditory neuropathy’ typically describes a form of hearing impairment independent of cochlear function and is characterized by the presence of abnormal afferent auditory neural conduction in the VIII nerve and auditory brainstem. Auditory neuropathy has been shown in a subset of mitochondrial diseases, including Friedreich ataxia,^8^ Leber’s hereditary optic neuropathy and *OPA1*-linked disorders.^9,10^ Lesions of the auditory pathways and auditory cortex often have a positive MRI with CNS involvement. This suggests that mitochondrial disease patients with central neurological manifestations may also have auditory neural dysfunction. For example, mitochondrial disorders with generalized neuropathic consequences and auditory neuropathy, including Leber’s hereditary optic neuropathy.^11^ Auditory neuropathies have been shown to disrupt perception in neural firing patterns resulting in a distorted representation of complex acoustic signals. The primary functional consequence of auditory neuropathy is impaired understanding of speech, particularly in background noise.^12^

The perceptual consequences of auditory neuropathy are distinct from those associated with sensory hearing loss. Cochlear level dysfunction typically affects sound detection thresholds, the ability to perceive changes in frequency/pitch and the discrimination of changes in intensity/loudness. Auditory neural defects, in contrast, disrupt neural firing patterns, leading to impaired neural representation of subtle temporal cues, which are critical for binaural processing (particularly sound localization) and speech understanding in background noise.^8^

Identifying the distinct pathology and mechanism of hearing impairment is critically important for clinical management of patients. Standard intervention options may not be appropriate for patients with solely neural or mixed cochlear/neural abnormalities. Conventional hearing aids function to amplify sound but do not correct for signal distortion or improve the processing of auditory temporal cues. As such, an individual with retro-cochlear deficits may be presented with a louder but equally distorted signal. Cochlear implant outcomes are dependent on the site of lesion, with variable results for sites central to the level of the spiral ganglion.^12^

Several aetiologies of hearing loss may also be accompanied by vestibular disturbance. The vestibular system is a key to numerous functions, from balance control and gaze stabilization to high-level functions involving spatial cognition. Impaired balance in adults with mitochondrial disease is often due to the involvement of multiple systems, with manifestations including ataxia, peripheral neuropathy, proximal myopathy and visual impairment, in addition to vestibular dysfunction. Mitochondrial disease therefore presents a model of multi-sensory neurological impairment, which has been investigated through cross-sectional cohorts^12,13^ and has led to a framework^14^ to improve differential diagnosis and hence provide clinically effective vestibular rehabilitation.

Vestibular dysfunction in individuals with m.3243A>G mitochondrial disease has been associated with the semi-circular canals, otolithic organs (utricles and saccules) and inferior vestibular nerve.^15,16^ However, the pathologic mechanisms of vestibular dysfunction in m.3243A>G mitochondrial disease are still not certain.

In this study, we aimed to investigate the prevalence and the functional consequences of auditory neuropathy, and its association with vestibular dysfunction, in individuals with m.3243A>G mitochondrial disease compared with an age and hearing level–matched control group. A deeper insight into audiological and vestibular characterization in patients with m.3243A>G mitochondrial disease at a mechanistic and functional level may help to direct potential clinical management and personalized treatment options that have the potential to improve patients’ quality of life.

## Materials and methods

This trial protocol was approved by the National Research *Ethics* Service Committee, the West of Scotland Research Ethics Committee 3 (Ref: 17/WS/0199) and the Human Research Ethics Committee of the Royal Victorian Eye and Ear Hospital, Melbourne, Australia (Ref: 07-747H-17). Informed consent was obtained from all participants prior to study entry. The study was performed in accordance with local laws, the International Conference on Harmonization Good Clinical Practice guidelines and the 1964 Declaration of Helsinki.

### Participants

Twelve adult participants (>18 years) confirmed by genetic testing to harbour the m.3243A>G pathogenic mtDNA variant and self-reported hearing and/or clinical evidence of balance or stability difficulties [Newcastle Mitochondrial Disease Scale for Adults (NMDAS) sub-score for hearing or gait stability between 2 and 4] were recruited from the NHS Highly Specialized Service for Rare Mitochondrial Disorders of Adults and Children, Newcastle upon Tyne Hospitals NHS Foundation Trust. Study exclusions were as follows: (i) participants not fluent in English, (ii) unable to produce a full range of speech sounds and (iii) current local outer or middle ear infection. Additionally, individuals with profound hearing loss (NMDAS sub-score 5), i.e. virtually no hearing despite a hearing aid (or those with a cochlear implant), were excluded given that (i) electrophysiologic testing would not provide diagnostic information and (ii) perceptual test stimuli would be inaudible.

Twelve control participants were also recruited from the University of Melbourne Audiology Clinic. As controls were individually matched for age at assessment (within 10 years), four-frequency average sound detection level [within 10 decibels (dB)] and sex, there were no significant group differences between age (m.3243A>G mitochondrial disease: 45.5 ± 16.3 years; controls: 46.6 ± 11.8 years, *P =* 0.54) and hearing level (m.3243A>G mitochondrial disease: 47.1 ± 21.5 dBHL; controls: 47.7 ± 25.4 dBHL, *P =* 0.81).

### Experimental procedures

#### Disease severity

Participants with the m.3243A>G variant underwent a physical examination, including an NMDAS^17^ assessment by a mitochondrial disease consultant neurologist. This semi-quantitative clinical rating scale designed specifically for all forms of adult mitochondrial disease was developed and tested with the aim of providing a validated and reproducible measurement of disease severity. Each question has a possible score from 0 to 5 with the higher the score, the more severe the disease.

#### Cognition

Addenbrooke’s cognitive examination III (ACE-III) was used to differentiate participants with and without cognitive impairment. ACE is composed of tests of attention/orientation, memory, fluency, language and visual perceptual/visuospatial ability.^18,19^

#### Audiometry

Sound detection thresholds were established to pure tone stimuli at octave frequencies across the audiometric range (between 250 Hz and 8 kHz). A four-frequency hearing level average was calculated based on hearing levels obtained at 0.5, 1, 2 and 4 kHz. Each ear was tested separately, and the ear with better detection thresholds was used for subsequent (monaural) auditory function assessments.

#### Cochlear electroacoustic function

Distortion product otoacoustic emissions (DPOAEs) were used to evaluate the mechanical function of the cochlear (outer) hair cells. The emission was elicited by two stimulus tones (ranging from 902 to 2566 Hz), presented as pairs with a frequency ratio (f2/f1) of 1.22. Stimuli were presented at 55 dBSPL (f2) and 65 dBSPL (f1). Average DPOAE amplitude (dB) was calculated based on response level at four test frequencies. Responses were considered present when the waveform repeatability was >50% and when DPOAE amplitude was ≥6 dB in at least three of the four frequencies.

#### Auditory neural function

Auditory brainstem responses (ABRs: electrophysiologic potentials from the VIII nerve and auditory brainstem) were recorded to 100 µs acoustic clicks presented at stimulus rates varying from 8 to 100 Hz. Stimulus presentation level was in the range of 90–100 dBnHL depending on the average hearing level of the participant. A minimum stimulus presentation level of 25 dB was maintained for each subject. EEG samples following 2000 stimuli were averaged to produce each test run, and two runs were obtained and compared to determine waveform repeatability. Analysis of the recordings was carried out independently by two experienced clinicians blinded to group status. These investigators determined response presence/absence; post-stimulus latency of Waves I, III and V and peak-to-peak amplitude of Waves I and V.

#### Auditory temporal processing

Auditory temporal resolution was assessed using an amplitude modulation (AM) detection task. The psychophysical protocol employed an adaptive, three-alternative, forced-choice task, which sought the minimum detectable depth of sinusoidal AM at two modulation rates: 10 and 150 Hz.^8^

#### Speech perception

Monaural speech perception testing was carried out using recorded consonant-nucleus-consonant (CNC) words presented via ER-4 insert phones to the test ear at 85 dBSPL (root-mean-square [RMS]). Each participant was assessed using two lists of 50 CNC words phonemically balanced to Australian English.^20^ One list was presented in quiet test conditions and the other with a competing noise (four-talker babble) presented at the same level as the speech (i.e. 0 dB signal-to-noise ratio). The participant imitated each stimulus word, and responses were phonetically transcribed, with the overall percentage of phonemes (speech sounds) correct calculated for each listening condition.

Binaural speech perception was assessed using the Listening in Spatialized Noise-Sentences test. The Listening in Spatialized Noise-Sentences test measures the participant’s ability to segregate a target speech signal from competing speech noise in a 3D auditory environment created by synthesizing the test stimuli with head-related transfer functions under headphones.^21^ The speech reception threshold [the signal-to-noise ratio required to identify 50% of the words in a target sentence (SRT)] was established in four conditions that vary in terms of the location of the noise source (0° versus 90° azimuth) and vocal quality of the speaker used to produce the target and background signals (same or different voice). The four conditions were DV90 (different voices spatially separated by 90°), SV90 (same voice separated by 90°), DV0 (different voices from same direction) and SV0 (same voice from same direction). An ‘advantage’ measure representing the dB benefit afforded by spatial cues was also calculated.^8^

#### Self-reported hearing disability

The Speech, Spatial and Qualities of Hearing Scale (SSQ) was used to assess participants’ self-reported hearing abilities across a variety of listening and communication scenarios in the everyday world.^22^ It has 49 items, which evaluate 3 listening domains: speech understanding, spatial hearing and qualities of sound.

#### Vestibular function and balance

The impact of dizziness on daily life was quantified by the 25-item self-report Dizziness Handicap Inventory (DHI) questionnaire. The items were sub-grouped into domains representing functional, physical and emotional impacts on disability.^23^

The modified Clinical Test of Sensory Interaction in Balance (mCTSIB) was used to quantify postural control under various sensory conditions.^24^ The test is designed to determine how well a participant uses sensory inputs when one or more sensory inputs are compromised.

The video head impulse test (vHIT) was used to assess high-frequency horizontal semi-circular canal (HSCC) function. The horizontal angular vestibulo-ocular reflex (VOR) gain was determined by measuring the ratio of eye movement in relation to an abrupt head rotation in the horizontal plane. Peak angular velocity ranged between 150 and 250°/s with a peak angular acceleration of 2000°/s^2^. Abnormal VOR gain was quantified using a cut-off of VOR gain <0.8, in combination with corrective saccades (covert or overt).

#### Statistical analyses

The target sample size of 24 participants (12 subjects with m.3243A>G mitochondrial disease and 12 matched control participants) was empirically determined to meet the study objectives. No formal hypothesis testing was planned. Group differences were explored using regression-based models with covariate adjustments for age and hearing level. Unadjusted comparisons were performed using paired *t*-tests. No imputation for missing values was included. Pearson correlation coefficient (*r*) was used to investigate relationships. Statistical significance was determined at *P* < 0.05. Data are summarized using descriptive statistics (e.g. mean, SD, 95% confidence interval).

## Results

### Clinical characteristics

Participant demographics and clinical characteristics are presented in Table 1. Half of the cohort with m.3243A>G mitochondrial disease (*n =* 6; 50%) had diabetes. Of those, four had subtle neuropathy (NMDAS 1 and 2); two additional (non-diabetic) participants also had subtle neuropathy. Abnormalities in gait stability and cerebellar ataxia were predominant (*n =* 8, respectively, both present in six participants).

**Table 1:** Summary demographics and clinical characteristics for participants with m.3243A>G mitochondrial disease

	Sex	Age (years)	Cognition	Cochlear emissions	Sound detection	Heteroplasmy (%)^d^			Disease burden	Clinical characteristics (NMDAS sub-scales scores, 0–5)									
			ACE-III^a^	DPOAE^b^	AHL (dB)^c^	Blood	Urine	Skeletal muscle	NMDAS total^e^	Hearing^f^	Diabetes^g^	Neuropathy^h^	Gait stability^i^	Cerebellar ataxia^j^	Myopathy^k^	Ptosis^l^	CPEO^m^	Migraines^n^	Vision^o^
m.3243A>G 01	F	18	72	Present	17.5	89	80	66	21.5	2	0	0	2	1	2	1	0	5	0
02	M	21	NP	Absent	50.0	100	92	N/A	28	2	0	1	2	0	3	0	0	5	0
03	F	24	85	Present	41.3	100	89	80	25.9	2	5	0	2	1	2	0	0	3	1
04	F	43	87	Absent	74.2	81	74	78	18.6	4	0	0	3	2	2	1	0	0	0
05	M	46	96	Absent	61.3	67	76	66	30.1	2	5	0	2	4	2	1	1	5	0
06	F	48	92	Absent	61.3	79	87	75	19.7	2	5	1	1	2	1	1	0	1	0
07	F	49	95	Present	21.2	26	27	52	19.7	0	0	0	3	2	2	1	0	1	2
08	M	54	93	Absent	60.0	59	83	67	45.6	3	5	2	3	2	2	3	0	0	1
09	F	55	87	Absent	73.8	67	77	67	32.1	3	5	1	1	1	2	0	0	2	2
10	F	58	95	Present	15.0	24	31	N/A	11.8	0	0	0	2	2	0	1	0	¾	1
11	F	64	96	Present	30.0	9	12	N/A	19.7	3	4	1	2	2	1	1	0	1	0
12	F	66	98	Absent	60.0	46	62	N/A	15.5	3	0	1	3	2	2	0	0	0	0
m.3243A>G, (*n =* 12), mean (SD)		45.5 (16.3)	90.5 (7.5)		47.1 (21.5)	62.3 (30.3)	65.8 (27.1)	68.9 (8.9)	24.0 (9.1)										
Control (*n =* 12), mean (SD)		46.6 (11.8)	93.5 (8.6)		47.7 (25.4)	N/A	N/A	N/A	N/A										

Demographics and clinical characteristics for each of the m.3243A>G mitochondrial disease participants. Group means (±standard deviation) are also shown for matched control participants.

AHL, audiometrically measured hearing level; CPEO, chronic progressive external ophthalmoplegia; DPOAE, distortion product otoacoustic emission, NP, not performed.

^a^ACE-III total score = sum of item scores. Maximum total score = 100 (18 for attention, 26 for memory, 14 for fluency, 26 for language and 16 for visuospatial); minimum score = 0. Higher scores indicate better cognitive functioning. Score cut-offs for dementia: <82; mild cognitive impairment: <88.

^b^DPOAE = DPOAE response present: ≥6 dB.

^c^AHL = four-frequency average hearing levels (0.5 Hz, 1 kHz, 2 kHz and 4 kHz) for better ear. Normal: ≤15 dBHL; mild degree hearing loss (21–40 dBHL); moderate hearing loss (41–70 dB); severe hearing loss (71–90 dB).

^d^Heteroplasmy levels for blood and urine were age and sex adjusted, respectively.

^e^NMDAS. Total = scaled score. Higher scores indicate greater disease severity.

^f^Hearing (with or without hearing aids): 2 = mild deafness, missing words in presence of background noise, fully corrected with hearing aids; 3 = moderate deafness, regularly requiring repetition, not fully corrected with hearing aids; 4 = severe deafness, poor hearing even with hearing aids.

^g^Diabetes mellitus: 0 = none; 4 = non–insulin-dependent diabetes mellitus (tablets); 5 = diabetes mellitus requiring insulin (irrespective of treatment at onset).

^h^Neuropathy: 0 = none; 1 = subtle sensory symptoms or areflexia; 2 = sensory impairment only (e.g. glove and stocking sensory loss).

^i^Gait stability: 1 = normal, occasional difficulties on turns, uneven ground or if required to balance on narrow base; 2 = reasonably steady, aware of impaired balance and occasionally off balance when walking; 3 = unsteady, always off balance when walking, occasional falls and gait steady with support of stick or person.

^j^Cerebellar ataxia: 0 = none; 1 = normal gait but hesitant heel-toe; 2 = reasonably steady gate, unable to maintain heel-toe walking or mild upper limb (UL) dysmetria; 3 = ataxic gait (but walks unaided) or UL intention tremor and past-pointing, unable to walk heel-toe—falls immediately; 4 = severe, gait grossly unsteady without support or UL ataxia sufficient to affect feeding.

^k^Myopathy: 0 = normal; 1 = minimal reduction in hip flexion and/or shoulder abduction only (Medical Research Council Score [MRC] 4+/5); 2 = mild but clear proximal weakness in hip flexion and shoulder abduction (MRC 4/5), minimal weakness in elbow flexion and knee extension (MRC 4+/5− both with joint at 90°); 3 = moderate proximal weakness including elbow flexion and knee extension (MRC 4/5 or 4−/5) or difficulty rising from a 90° squat.

^l^Ptosis: 0 = none; 1 = mild, not obscuring either pupil; 3 = bilateral, obscuring < 1/3 or unilateral, obscuring > 1/3 of pupil or prior unilateral surgery.

^m^CPEO: 0 = none; 1 = some restriction of eye movement in any direction. Abduction complete.

^n^Migraine headaches: 0 = no past history; 1 = asymptomatic but past history; 2 = 1 day per month; 3 = 2 days per month; 5 = ≥4 days per month.

^o^Vision: 0 = normal; 1 = no functional impairment but aware of worsened acuities; 2 = mild (difficulty with small print/text on the television).

Average heteroplasmy levels for age-adjusted blood, sex-adjusted urine and skeletal muscle were 62.3 ± 30.3, 65.8 ± 27.1 and 68.9 ± 8.9%, respectively.

Cognitive function assessed by total ACE-III score was not significantly different between groups (m.3243A>G mitochondrial disease: 90.5 ± 7.5; controls: 93.5 ± 8.6, *P* > 0.05). However, four participants with m.3243A>G mitochondrial disease (36%) scored below the cut-off score for mild cognitive impairment or dementia^19,25^ (Table 1) compared with two control participants (18%; Supplementary Table 1).

### Sound detection levels

Hearing acuity varied considerably across m.3243A>G mitochondrial disease participants, with four-frequency average hearing values in the better ear ranging from normal to severe levels (Table 1). Audiometric configuration was variable, but most individuals showed flat hearing losses and broadly equivalent sound detection across the speech frequency range. Hearing levels in most cases were symmetrical (≤10 dB inter-aural difference) with similar sound detection thresholds for each ear. In each group, 1 of 12 participants had normal sound detection (≤15 dBHL). The degree of hearing loss in the remaining 11 participants of each group was as follows: mild, *n =* 3 (21–40 dBHL); moderate, *n =* 6 (41–70 dB) and severe, *n =* 2 (71–90 dB).

### Otoacoustic emissions

Normal cochlear outer hair cell function was indicated by the presence of a repeatable otoacoustic emission in each m.3243A>G mitochondrial disease participant with normal or near-normal sound detection thresholds (*n =* 4; Table 1). Seven of the eight remaining m.3243A>G mitochondrial disease participants with significant hearing loss (four-frequency average >40 dBHL) showed no DPOAE response (consistent with the presence of cochlear pathology), but one individual presented with a recordable emission despite the presence of moderate-degree hearing loss (Table 1). Among control participants, DPOAEs were present in the four individuals with normal or near-normal sound detection and absent in each case with significant hearing loss (Supplementary Table 1).

### Neuro-audiology

Six of 12 individuals with the m.3243A>G variant showed absent or clinically abnormal ABRs. Four of these had unrecordable responses to acoustic stimuli presented at the standard clinical rate of 33 clicks per second (Table 2). A further two participants showed repeatable responses, but clinically abnormal neural conduction velocities (ABR Wave I–V inter-peak latency >4.4 ms.^26^ In contrast, each control participant showed repeatable ABRs and neural conduction speeds within normal limits (Supplementary Table 1). As such, the rate of ABR abnormality observed for the m.3243A>G mitochondrial disease group [*n =* 6/12 (50%)] was significantly higher than for the hearing level–matched controls [*n =* 0/12 (0%); *P =* 0.005].

**Table 2:** ABR findings for individuals with m.3243A>G mitochondrial disease

	Absolute response latencies (ms)			Inter-peak latencies (ms)			Amplitude (μV)		
	Wave I	Wave III	Wave V	I–III	III–V	I–V^a^	V/I ratio	Maximum rate^b^	ABR result
m.3243A>G 01	1.50	3.59	5.38	2.08	1.79	3.88	0.52	100	Normal
02	1.84	4.21	5.92	2.38	1.71	4.08	0.17	100	Normal
03	1.57	4.17	6.00	2.60	1.83	4.43	0.35	33	Prolonged I–V. Rate effect
04	^d^	^d^	^d^	^d^	^d^	^d^	^d^	0	Absent
05	^d^	^d^	^d^	^d^	^d^	^d^	^d^	0	Absent
06	^d^	^d^	^d^	^d^	^d^	^d^	^d^	0	Absent
07	1.46	3.63	5.63	2.17	2.10	4.17	0.19	100	Normal
08	^d^	^d^	^d^	^d^	^d^	^d^	^d^	8	Absent (≥33 Hz). Rate effect
09	1.71	4.17	5.84	2.46	1.67	4.13	0.31	75	Normal
10	1.67	3.75	5.5	2.08	1.75	3.83	0.32	100	Normal
11	1.46	3.85	5.38	2.39	1.53	3.92	0.14	100	Normal
12	1.38	3.78	6.09	2.41	2.30	4.71	0.11	33	Prolonged I–V. Rate effect
m.3243A>G (*n =* 8), mean (SD)	1.57 (0.15)	3.89 (0.25)	5.72 (0.28)	2.32 (0.19)	1.83 (0.25)	4.14 (0.30)	0.26 (0.14)	54.1 (45.5)	
Control (*n =* 12), mean (SD)	1.59 (0.21)	3.80 (0.23)	5.64 (0.31)	2.21 (0.16)	1.80 (0.21)	4.05 (0.21)	0.47 (0.19)	95.8 (9.7)	Normal—all control participants
m.3243A>G/control difference, mean (SD)	−0.02 (0.22)	0.09 (0.24)	0.07 (0.39)	0.11 (0.19)	0.04 (0.37)	0.09 (0.29)	−0.21 (0.14)	−41.8 (41.3)	
95% CI for paired difference	−0.20, 0.17	−0.11,0.29	−0.25, 0.40	−0.05, 0.26	−0.27, 0.35	−0.15, 0.34	−0.32, −0.09	−68.0, −15.5	
*P*-value	0.82	0.32	0.61	0.15	0.79	0.41	**0**.**005**	**0.005**	
*P*-value adjusted^c^	0.83	0.19	0.50	**0**.**04**	0.96	0.34	**0**.**03**	**0.002**	

ABR latencies and amplitudes for each of the m.3243A>G mitochondrial disease participants. Group means (±standard deviation) are also shown for matched control participants. Bold represents significant differences between m.3243A>G and controls, *P* < 0.05. Response latencies are expressed in milliseconds, and amplitudes are expressed in microvolts (µV).

^a^Abnormal I–V inter-peak latency, i.e. conduction time between Wave I and V: >4.4 ms.

^b^Maximum rate is the highest stimulus presentation rate [hertz (Hz)] at which an ABR could be identified.

^c^Adjusted for age and hearing level.

^d^Waveform absence.

ABR latencies (for the eight m.3243A>G mitochondrial disease individuals who had recordable potentials at 33 Hz) were not significantly different from those of the matched controls (Table 2). Response amplitude (Wave V/I ratio) was, however, significantly lower in participants with the m.3243A>G variant (0.26 ± 0.14) when compared with controls (0.47 ± 0.19, *P =* 0.005).

**Figure 1:**
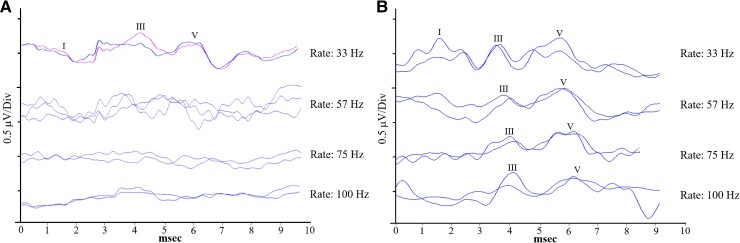
**ABR in a participant with m.3243A>G mitochondrial disease versus a matched control.** ABR in a participant with m.3243A>G mitochondrial disease (#12) (**A**) compared with her matched control (**B**), in response to acoustic click stimuli at presentation rates ranging from 33 to 100 Hz. The annotations ‘I’, ‘III’ and ‘V’ represent the positive peaks in the ABR waveform complex. The *x*-axis shows the post-stimulus latency in milliseconds.

Auditory brainstem potentials for participants with the m.3243A>G variant were abnormally sensitive to increases in stimulus rate. Figure 1 shows an example of this effect where a repeatable ABR was elicited in the m.3243A>G mitochondrial disease participant #12 to acoustic clicks at 33 Hz but was unrecordable to the same stimulus at higher rates. The maximum presentation rate at which a repeatable ABR waveform could be identified was significantly lower in participants with m.3243A>G mitochondrial disease compared with controls (m.3243A>G mitochondrial disease: 54.1 ± 45.5 Hz; controls: 95.8 ± 9.7 Hz, *P =* 0.005; Table 2, Supplementary Table 1). An ABR rate effect was determined by using a mean ± 2 SD range from the control participants. Three individuals with the m.3243A>G variant were identified with a recordable ABR maximum rate outside this range (calculated as: 76.4–115.3; Table 2).

### Auditory temporal resolution

Perception of rapidly occurring acoustic changes was impaired in participants with m.3243A>G mitochondrial disease. While detection thresholds for low-rate (i.e. 10 Hz) amplitude modulations showed no group difference (m.3243A>G mitochondrial disease: −16.0 ± 6.5 dB; controls: −19.8 ± 2.0 dB, *P =* 0.062), participants with the m.3243A>G variant required a significantly greater depth of modulation to identify the amplitude variation for high-rate (i.e. 150 Hz) AM (mitochondrial disease: −11.3 ± 6.2 dB; controls: −17.2 ± 3.1 dB, *P =* 0.005; Table 3, Supplementary Table 2).

**Table 3:** Summary of temporal processing, speech perception and self-reported hearing disability for participants with m.3243A>G mitochondrial disease versus matched control participant

	Temporal resolution		Monaural speech perception		Binaural speech perception in noise					Self-reported hearing disability			
	AM detection (dB)^a^ (*n* = 11)		CNC words (%)^b^ (*n* = 12)		LISN-S test, speech reception threshold (dB)^c^ (*n* = 12)					SSQ^d^ (*n* = 11)			
	10 Hz	150 Hz	Quiet	Noise	DV90°	SV90°	DV0°	SV0°	Spatial adv	Speech	Spatial	Quality	Average
m.3243A>G	−16.0 (6.5)	−11.3 (6.2)	75.6 (30.1)	27.7 (21.1)	−2.4 (7.8)	−1.7 (7.2)	0.1 (2.8)	2.1 (2.3)	3.8 (5.2)	4.6 (1.9)	5.8 (2.2)	6.5 (2.3)	5.6 (1.8)
Control	−19.8 (2.0)	−17.2 (3.1)	80.2 (11.2)	41.8 (14.0)	−7.0 (7.8)	−6.0 (7.7)	−1.7 (4.4)	1.7 (4.1)	7.7 (4.5)	5.8 (2.1)	5.3 (2.6)	5.9 (4.5)	5.6 (2.1)
m.3243A>G/control difference	3.8 (6.0)	5.9 (5.6)	−4.6 (26.5)	−14.1 (15.3)	4.6 (6.3)	4.3 (5.4)	1.8 (4.1)	0.4 (4.1)	3.9 (2.8)	−1.1 (2.4)	0.5 (2.8)	0.6 (3.3)	−0.0 (2.3)
95% CI for paired difference	−0.2, 7.9	2.2, 9.7	−21.5, 12.2	−23.8, −4.4	0.6, 8.6	0.9, 7.8	−0.8, 4.4	−2.2, 3.0	−5.7, −2.1	−2.7, 0.4	−1.4, 2.4	−1.6, 2.8	−1.5, 1.5
*P*-value	0.062	**0.005**	0.558	**0.008**	**0.028**	**0.019**	0.15	0.72	**0.001**	0.14	0.56	0.54	0.99
*P*-value (adjusted)^e^	0.084	**0.002**	0.497	**0.003**	**0.002**	**0.003**	0.059	0.60	**0.001**	0.182	0.60	0.55	0.55

Temporal processing, speech perception and self-reported hearing disability for participants with m.3243A>G mitochondrial disease compared with matched control participants. Data are means (±standard deviation). Bold represents significant differences between m.3243A>G and controls, *P* < 0.05.

DV90°, different voices (target sentence and background) separated by 90°; DV0°, different voices, same direction; LISN-S, Listening in Spatialized Noise-Sentences test; spatial adv, spatial advantage, improvement in speech reception threshold when the target speech and noise are spatially separated; SV90°, same voice separated by 90°; SV0°, same voice, same direction.

^a^AM depth threshold = smallest detectable modulation depth (dB) in a burst of white noise.

^b^CNC words test = phoneme score (% correct). Quiet: abnormal: <89.5%; noise (0 dB SNR): abnormal: <36.2%.

^c^Speech reception threshold = the signal-to-noise ratio required to identify 50% of the words in a target sentence.

^d^SSQ: maximum average score = 10; minimum score = 0. Higher scores reflect greater ability (less disability).

^e^Adjusted for age and hearing level.

### Speech perception

#### Monaural

Open-set speech perception ability was diminished in mitochondrial disease participants. While there was no group difference in the percentage of phonemes discriminated in the quiet listening condition (m.3243A>G mitochondrial disease: 75.6 ± 30.1%; controls: 80.2 ± 11.2%, *P =* 0.558), perception in the presence of background noise was significantly poorer for participants with the m.3243A>G variant (m.3243A>G mitochondrial disease: 27.7 ± 21.1%; controls: 41.8 ± 14.0%, *P =* 0.008; Table 3, Supplementary Table 2).

#### Binaural

Binaural speech perception was also impaired in m.3243A>G mitochondrial disease listeners. Mean SRT for conditions replicating everyday listening (where target sentences and noise emanate from different directions) was >4 dB higher in participants with m.3243A>G mitochondrial disease, indicating that noise levels needed to be significantly lower in this cohort for adequate perception (DV90: m.3243A>G mitochondrial disease: −2.4 ± 7.8 dB; controls: −7.0 ± 7.8 dB, *P =* 0.028 and SV90: m.3243A>G mitochondrial disease: −1.7 ± 7.2 dB; controls: 6.0 ± 7.7 dB, *P =* 0.019; Table 3, Supplementary Table 2). In contrast, there were no group differences for listening conditions in which target speech and noise were presented from the same direction (DV0: m.3243A>G mitochondrial disease: 0.1 ± 2.8 dB; controls: −1.7 ± 4.4 dB, *P =* 0.15 and SV0: m.3243A>G mitochondrial disease: 2.1 ± 2.3 dB; controls: 1.7 ± 4.1 dB, *P =* 0.72; Table 3, Supplementary Table 2).

This result pattern (abnormal perception for spatially separated acoustic signals) reflects an impaired ability to localize sound sources to improve speech understanding in noise. As such, the mean improvement in SRT (spatial advantage) when target and background noise were separated by 90° was 3.9 dB poorer for participants with m.3243A>G mitochondrial disease than their matched counterparts (m.3243A>G mitochondrial disease: 3.8 ± 5.2 dB; controls: 7.7 ± 4.5 dB, *P =* 0.001; Table 3, Supplementary Table 2).

### Self-reported hearing disability

Individuals with m.3243A>G mitochondrial disease reported similar degrees of everyday speech understanding and communication difficulty to their matched peers. No significant group differences were found for any SSQ domains (speech perception, spatial hearing and sound quality) or the average across domains (*P =* 0.99; Table 3, Supplementary Table 2).

### Balance and vestibular function

#### Self-reported dizziness

All 11 participants with m.3243A>G mitochondrial disease who completed the self-reported DHI perceived a degree of dizziness, from mild to severe handicap (range: 20–72), with moderate-to-severe dizziness self-reported in 6 participants. In contrast, only three control participants reported mild dizziness, with the mean total DHI score significantly higher in m.3243A>G mitochondrial disease participants compared with matched controls (*P =* 0.001; Table 4, Supplementary Table 3).

**Table 4:** Summary of vestibular function, balance and self-reported dizziness for participants with m.3243A>G mitochondrial disease versus matched control participants

	Vestibular	Self-reported dizziness (DHI)^c^				mCTSIB^d^
	VOR gain^a,b^	Physical	Emotional	Functional	Total	(s)
m.3243A>G (*n* = 11)^a^	0.71 (0.20)	12.2 (4.0)	12.4 (7.9)	16.5 (9.8)	41.1 (18.4)	87.3 (27.8)
Control (*n* = 11)	0.91 (0.16)	4.4 (5.2)	1.5 (2.6)	3.3 (3.5)	9.1 (10.8)	117.2 (6.3)
m.3243A>G/control difference	−0.19 (0.19)	7.4 (8.0)	11.8 (8.0)	14.2 (10.6)	35.8 (22.2)	30.7 (29.0)
95% CI for paired difference	−0.33, −0.06	1.7, 13.1	6.1, 17.5	6.6, 21.8	17.5, 49.2	9.9. 51.5
*P*-value	**0.011**	**0.017**	**0.001**	**0.002**	**0.001**	**0.009**
*P*-value (adjusted)^e^	**0.004**	**0.0012**	**0.0006**	**0.0004**	**<0.001**	**0.003**

Vestibular function, self-reported dizziness and balance for participants with m.3243A>G mitochondrial disease compared with matched control participants. Data are means (±standard deviation). Bold represents significant differences between m.3243A>G and controls, *P* < 0.05.

^a^Number of patients with mitochondrial disease completing vHIT: *n* = 10. Two patients were unable to complete the test due to experiencing unsteadiness, double vision (*n* = 1) or dizziness (*n* = 1).

^b^VOR gain abnormal: <0.80.

^c^DHI total score = sum of item scores. Maximum total score = 100 (28 for physical, 36 for emotional and 36 for functional) minimum score = 0. Higher scores indicate greater perceived disability. Handicap cut-off scores: mild = 16–34; moderate = 36–52; severe ≥ 54.

^d^mCTSIB: time maintaining balance across 4 conditions, with 30 s the maximum duration for each condition. Condition 1 = eyes open, firm surface; Condition 2 = eyes closed, firm surface; Condition 3 = eyes open, foam surface; Condition 4 = eyes closed, foam surface. Total maximum time across all 4 conditions = 120 s.

^e^Adjusted for age and hearing level.

#### Balance

Ten participants from each group completed the mCTSIB. The mean standing balance time achieved across the conditions in the mCTSIB test was significantly lower in participants with m.3243A>G mitochondrial disease when compared with matched controls (m.3243A>G mitochondrial disease: 87.3 ± 27.8 s; controls: 117.2 ± 6.3 s, *P* = 0.009); Table 4). A total of 8 of 10 (80%) m.3243A>G mitochondrial disease participants were unable to maintain upright posture when somatosensory information was reduced and vision was occluded [Condition 4 (C4), eyes closed/foam surface], indicating failure of the vestibular inputs required to maintain postural control. Of the eight participants unable to maintain posture in C4, half of the cases (#04, 05, 07 and 12) were also unable to maintain balance under C3 (eyes open, foam surface), indicating poor use of the visual system to compensate. Visual impairment was not likely to explain this loss of balance, with mild impairment (vision NMDAS score of 2) present in one of four participants. Additionally, occlusion of vision was not present, and participants were presented with either no ptosis (*n* = 1) or mild ptosis [i.e. not obscuring either pupil (*n* = 3)]. While peripheral neuropathy was not prominent (subtle in one participant), all four participants failing to maintain upright posture under C3 and C4 had a combination of some muscle weakness (myopathy NMDAS score of 2) combined with cerebellar ataxia and balance instability during gait (Table 5).

**Table 5:** Vestibular function, balance and self-reported dizziness (and associated clinical characteristics) for individuals with m.3243A>G mitochondrial disease

		mCTSIB^b^	VOR^c^					Dizziness	Clinical characteristics (NMDAS sub-scales scores, 0–5)^e^					
	AHL (dB)^a^	(s)	Comments	Left	Right	Test ear	vHIT comments	DHI total^d^	Vision^f^	Ptosis^g^	Neuropathy^h^	Gait stability^i^	Cerebellar ataxia^j^	Myopathy^k^
m.3243A>G 01	17.5 (mild)	120	Normal	0.88	0.93	0.88	Normal HSCC (bilateral)	20 (mild)	0	1	0	2	1	2
02	50.0 (moderate)	NP	NP	0.93	0.96	0.96	Normal HSCC (bilateral)	NP	0	0	1	2	0	3
03	41.3 (moderate)	96	Abnormal condition: 4	Incomplete—double vision/unsteadiness				30 (mild)	1	0	0	2	1	2
04	74.2 (severe)	30	Abnormal condition: 4, 3, 2	0.75	0.71	0.71	Abnormal—corrective saccades	60 (severe)	0	1	0	3	2	2
05	61.3 (moderate)	83	Abnormal condition: 4, 3	0.61	0.67	0.67	HSCC dysfunction (bilateral)	44 (moderate)	0	1	0	2	4	2
06	61.3 (moderate)	92	Abnormal condition: 4	0.86	0.71	0.71	HSCC dysfunction (right)	28 (mild)	0	1	1	1	2	1
07	21.2 (mild)	56	Abnormal condition: 4, 3, 2	0.91	0.91	0.91	Normal HSCC (bilateral)	64 (severe)	2	1	0	3	2	2
08	60.0 (moderate)	90	Abnormal condition: 4	0.61	0.67	0.67	HSCC dysfunction (bilateral)	72 (severe)	1	3	2	3	2	2
09	73.8 (severe)	120	Normal	0.26	0.31	0.26	HSCC dysfunction (bilateral)	36 (moderate)	2	0	1	1	1	2
10	15.0 (normal)	65	Abnormal condition: 4, 2	Incomplete—patient became dizzy				52 (moderate)	1	1	0	2	2	0
11	30.0 (mild)	113	Normal	0.86	1.14	0.86	Normal HSCC (bilateral)	26 (mild)	0	1	1	2	2	1
12	60.0 (moderate)	93	Abnormal condition: 4, 3	0.54	0.61	0.54	HSCC dysfunction (bilateral)	20 (mild)	0	0	1	3	2	2

Sound detection, vestibular function, balance and self-reported dizziness for each of the m.3243A>G mitochondrial disease participants.

AHL, audiometrically measured hearing level, NP, not performed.

^a^AHL = four-frequency average hearing levels (0.5 Hz, 1 kHz, 2 kHz and 4 kHz) for better ear. Normal: ≤15 dBHL; mild degree hearing loss (21–40 dBHL); moderate hearing loss (41–70 dB); severe hearing loss (71–90 dB).

^b^mCTSIB: time maintaining balance across four conditions, with 30 s the maximum duration for each condition. Abnormal = if there was more than one loss of balance across all trials. Condition 1 = eyes open, firm surface; Condition 2 = eyes closed, firm surface; Condition 3 = eyes open, foam surface; Condition 4 = eyes closed, foam surface.

^c^VOR gain abnormal < 0.80.

^d^DHI Total score = sum of item scores. Maximum total score = 100 (28 for physical, 36 for emotional and 36 for functional), minimum score = 0. Higher scores indicate greater perceived disability. Handicap cut-off scores: mild = 16–34; moderate = 36–52; severe ≥ 54.

^e^NMDAS. Total = scaled score. Higher scores indicate greater disease severity.

^f^Vision: 0 = normal; 1 = no functional impairment but aware of worsened acuities; 2 = mild (difficulty with small print/text on the television).

^g^Ptosis: 0 = none; 1 = mild, not obscuring either pupil; 3 = bilateral, obscuring < 1/3 or unilateral, obscuring > 1/3 of pupil or prior unilateral surgery.

^h^Neuropathy: 0 = none; 1 = subtle sensory symptoms or areflexia; 2 = sensory impairment only (e.g. glove and stocking sensory loss).

^i^Gait stability: 1 = normal, occasional difficulties on turns, uneven ground or if required to balance on narrow base; 2 = reasonably steady, aware of impaired balance, occasionally off balance when walking; 3 = unsteady, always off balance when walking, occasional falls and gait steady with support of stick or person.

^j^Cerebellar ataxia: 0 = none; 1 = normal gait but hesitant heel-toe; 2 = reasonably steady gate, unable to maintain heel-toe walking or mild upper limb (UL) dysmetria; 3 = ataxic gait (but walks unaided) or UL intention tremor and past-pointing, unable to walk heel-toe—falls immediately; 4 = severe, gait grossly unsteady without support or UL ataxia sufficient to affect feeding.

^k^Myopathy: 0 = normal; 1 = minimal reduction in hip flexion and/or shoulder abduction only (MRC 4+/5); 2 = mild but clear proximal weakness in hip flexion and shoulder abduction (MRC 4/5), minimal weakness in elbow flexion and knee extension (MRC 4+/5− both with joint at 90°); 3 = moderate proximal weakness including elbow flexion and knee extension (MRC 4/5 or 4−/5) or difficulty rising from a 90° squat.

One participant with the m.3243A>G variant indicated an abnormal visual dependency for postural control, failing to maintain posture under both visual occluded conditions (C4 and C2), but maintained balance in C3 (eyes open, foam surface). While this participant did not have any other clinical features that would affect the ability to use somatosensory inputs to maintain balance (i.e. peripheral neuropathy), a moderate degree of self-reported dizziness (DHI) was suggestive of vestibular dysfunction. This participant was unable to complete vHIT due to dizziness. In comparison, all control participants maintained postural control through to C3, with 8 of 10 participants completing C4 (Supplementary Table 3).

#### Vestibular function

Two m.3243A>G mitochondrial disease participants were unable to complete the vestibular function test (vHIT), either becoming dizzy during the assessment (*n =* 1) or experiencing double vision/unsteadiness (*n =* 1). Vestibular function (VOR gain) data were therefore available for 10 participants with m.3243A>G mitochondrial disease (and their matched controls). Of these, m.3243A>G mitochondrial disease participants had significantly poorer vestibular function (VOR gain) compared with controls (*P =* 0.011; Table 4). Six of the 10 participants with the m.3243A>G variant (60%) had clinically abnormal vestibulo-ocular reflexes, characterized by reduced VOR gain and corrective saccades (*n =* 6), HSCC dysfunction (right, *n =* 1) and HSCC dysfunction (bilateral, *n =* 5) (Table 5). In comparison, two (17%) of the matched controls were deemed abnormal [HSCC dysfunction (bilateral, *n =* 1) and HSCC dysfunction (left, *n =* 1); Supplementary Table 3].

### Associations between auditory and vestibular measures

Pearson correlations were used to investigate associations between electrophysiologic stimulus presentation rate (maximum rate with a recordable ABR), auditory temporal processing [high-rate (150 Hz) modulation detection], binaural speech perception (spatial advantage) and vestibular function (VOR gain; Table 6).

**Table 6:** Pairwise Pearson *r* correlations showing the association between auditory and vestibular measures

Measure 1	Measure 2	Correlation	95% CI	*P*-value
ABR (maximum rate) (Hz)	AM 150 Hz	−0.77	(−0.94, −0.31)	**0**.**006**
ABR (maximum rate) (Hz)	Binaural processing (spatial advantage)	0.70	(0.20, 0.91)	**0**.**012**
ABR (maximum rate) (Hz)	Vestibular function (VOR)	0.37	(−0.33, 0.81)	0.291
AM 150 Hz	Binaural processing (spatial advantage)	−0.71	(−0.92, −0.20)	**0**.**014**
AM 150 Hz	Vestibular function (VOR)	−0.82	(−0.96, −0.35)	**0**.**007**
Binaural processing (spatial advantage)	Vestibular function (VOR)	0.63	(0.00, −0.09)	**0**.**041**

Bold represents a significant correlation, *P* < 0.05.

Individuals presenting with the lowest maximum ABR rates also presented with the most impaired temporal resolution (*r* = −0.77, *P =* 0.006) and poorest speech perception (ABR rate: *r* = −0.70, *P =* 0.012). Both temporal resolution and speech perception were correlated with vestibular function (AM = 150 Hz: *r* = −0.82, *P =* 0.007; spatial advantage: *r* = 0.63, *P =* 0.041). Vestibular function was not correlated with the auditory electrophysiologic variables (*P* > 0.05).

In m.3243A>G mitochondrial disease participants, there were no correlations found between any of the clinical characteristics [disease severity (total NMDAS)], clinical features [sub-scales of NMDAS: hearing, diabetes, neuropathy, gait stability, cerebella ataxia, myopathy, vision, ptosis, chronic progressive external ophthalmoplegia and migraines], hetroplasmy [blood (age adjusted) and urine (sex adjusted)], cognition (ACE-III) and any outcome measures, including hearing level, temporal resolution, speech perception, subjective hearing disability, dizziness handicap, balance and vestibular function (*P* > 0.05).

## Discussion

This study revealed central auditory pathway abnormalities and perceptual deficits out of proportion with degree of peripheral hearing loss in adults with m.3243A>G mitochondrial disease. Furthermore, each participant reported some degree of dizziness handicap, and most showed evidence of vestibular dysfunction. As such, our group of patients harbouring the m.3243A>G pathogenic variant resemble those described previously with other mitochondrial disorders. Hearing and balance abnormalities associated with axonopathy in the vestibulocochlear nerve have, for example, been reported for a high proportion of patients with late-stage Friedreich ataxia.^27,28^ Similarly, auditory neuropathies are common in individuals with mitochondrial mutations underlying Leber’s hereditary optic neuropathy^11^ and Autosomal Dominant Optic Atrophy associated with *OPAI* pathogenic variant.^10^

ABRs were abnormal in a high proportion (50%) of participants with m.3243A>G mitochondrial disease. Four individuals showed absent ABR responses. In each of these cases, participants presented with moderate-to-severe hearing loss in the test ear (60.0–74.25 dBHL), which represented a stimulus sensation level of only 25–40 dB at maximum presentation levels. This sensation level was, however, sufficient to elicit repeatable ABRs in each of the hearing level–matched control participants.

One of the participants with the m.3243A>G variant who showed absent ABR met the clinical definition of auditory neuropathy, presenting with no recordable VIII nerve/brainstem potentials despite the presence of pre-neural responses (DPOAE) from the cochlear (outer) hair cells. ABR absence in these individuals offers no insight into particular pathological mechanisms involved in m.3243A>G-related mitochondrial disease. The response pattern observed for participants with recordable ABRs do, however, suggest some form of demyelinating process. Two individuals presented with prolonged neural conduction, particularly between VIII nerve and cochlear nucleus (ABR Wave I–III inter-peak latency). Furthermore, across all of the m.3243A>G mitochondrial disease participants with recordable ABRs, response amplitudes were significantly lower and sensitive to high-rate stimulation, significantly higher than for matched controls.

Demyelination increases membrane capacitance of a neuron and lowers resistance, thereby delaying excitation and reducing the velocity of action potential propagation.^29-33^ Demyelination may also affect the amplitude of the evoked potential in a number of ways. First, variable demyelination may lead to dyssynchrony if individual fibres are differentially demyelinated over different distances.^34^ Scalp-recorded ABRs are extracted from within the overall EEG via an averaging process^35^ and, as such, are extremely sensitive to temporal fluctuations in the neural response. As a result, timing variations as little as 0.5 ms may mean that some evoked activity does not add constructively to the averaged potential.^36^ Second, reduced evoked potential amplitudes may arise as a result of ‘conduction block’ where decreased membrane resistance short circuits the spread of the potential along the axon. In such cases, repetitive activation of demyelinated fibres results in a progressive increase in the conduction time of the action potential and may lead to an intermittent or total block in its propagation.^32,37^ Finally, significant demyelination may result in a secondary loss of nerve fibres (deafferentiation) reducing the population of axons available to contribute to the averaged response.^36^ Literature supports the notion that mitochondrial disease and dysfunction cause demyelination; studies report a significant downregulation of transcripts involved in myelination in several mitochondrial diseases.^38^ The pathological mechanisms underlying auditory dysfunction vary between mitochondrial diseases, indicated by electrophysiological findings relative to controls. For example, patients with Friedreich ataxia (FRDA) have a reduced ABR Wave V/I response amplitude but a normal neural conduction latency, consistent with impaired neural function due to auditory nerve axonal degeneration, but not demyelination.^8,27^ In patients harbouring the *OPA1* mutation, auditory dysfunction is likely caused by degeneration of the unmyelinated portion of the auditory nerve terminal dendrites within the cochlea. In more advanced stages of the disease, both demyelination and axonal loss of the entire auditory nerve may occur.^10^ In individuals with mutations associated with Leber’s hereditary optic neuropathy (m.11778G>A, m.14484T>C, m.14482 C>G and m.3460G>A), latencies for Waves III and V are significantly delayed, indicative of a demyelinating process in the auditory nerve and auditory brainstem (or possibly secondary demyelination following the loss of auditory nerve fibres).^11^

Interestingly, four of the six participants harbouring the m.3243A>G variant with ABR testing abnormalities presented with insulin-dependent diabetes, suggesting a potential contributing factor. Rance *et al*.^39^ has shown similar ABR results in individuals with Type 1 diabetes mellitus reporting prolonged neural conduction, attenuated response amplitudes and a lower maximum stimulus presentation rate able to elicit an ABR (electrophysiologic rate sensitivity). While there was no correlation between diabetes mellitus and any of the auditory/vestibular outcomes, this may reflect the small sample size.

In line with a rate effect observed in the ABR results, participants with the m.3243A>G variant also demonstrated an impaired ability to perceive rapidly occurring acoustic changes. While identification of sinusoidal AM over a low rate (10 Hz) was unaffected, detection of high-rate (150 Hz) amplitude variation was significantly impaired, suggesting that the central auditory pathways of participants with m.3243A>G-related mitochondrial disease were less able to encode rapid acoustic changes. Deficits in temporal resolution are a commonly reported feature of auditory neuropathies and have been described in patient populations with axonal and demyelinating neuropathy,^40^ autism spectrum disorder,^41,42^ diabetic neuropathy^39^ and neurofibromatosis.^43^

The functional result of disrupted auditory temporal processing is impaired discrimination of speech. In optimal (quiet) conditions, affected listeners struggle to differentiate speech sounds that differ only in their temporal characteristics including vowel length and consonant voicing.^27^ Furthermore, individuals with temporal processing deficit show particular difficulty in understanding speech in background noise. This may be the result of impaired ‘gap listening’ where disruption of the neural code means that the listener is less able to use brief quiet periods occurring in the background noise to access the embedded speech signal.^42^ In this study, monaural speech perception in quiet for listeners with the m.3243A>G variant was equivalent to that of matched sensory hearing loss controls, but perception in the presence of a competing noise was significantly poorer. Participants with the m.3243A>G variant on average could only identify approximately one in four speech sounds in background noise levels typical of everyday communication situations (0 dBSNR) which is insufficient for understanding of conversational speech.^8,36,44^

Neural disruption of binaural processing affects speech perception in noise as temporal distortion of the neural code for each ear means that they cannot be effectively combined in the central pathways. As a result, the listener is less able to localize sound sources and efficiently differentiate between target speech and background noise when they emanate from different directions.^45^ This process, known as ‘spatial streaming’, is contingent on the perception of subtle inter-aural cues, particularly the brief (≈50 µsec) timing differences that arise when sound reaches one ear before the other. In individuals with normal binaural processing, spatial streaming affords a 10 dB + release from the masking effects of background noise.^21^ In listeners with disrupted temporal processing, this phenomenon is diminished, and the degree to which it is reduced is correlated with degree of temporal resolution abnormality.^8,12^

Many of the participants with the m.3243A>G variant in the present study showed impaired speech perception in noise as a result of impaired spatial streaming. Nine of the 12 participants showed clinically abnormal spatial processing ability and speech reception thresholds (the signal-to-noise ratio required for the listener to identify 50% of the words in target sentences) for listening conditions where the speech and noise sources were spatially separated by 90° azimuth. Speech reception thresholds were significantly higher (≈4 dB) in participants with m.3243A>G variant than matched controls in both spatially separated listening conditions, indicating that for many individuals with m.3243A>G mitochondrial disease, everyday listening environments are perceived as significantly noisier. This degree of impairment has been reported to affect academic performance,^46^ cognitive function and physiologic stress levels.^47,48^ Importantly, speech perception in noise for m.3243A>G mitochondrial disease participants in listening conditions without binaural difference cues (i.e. where speech and noise emanated from the same direction) was relatively unimpaired. This finding indicates that other factors, such as cognitive impairments,^49,50^ which have been reported in mitochondrial disease, were not affecting the results and confirmed that the observed auditory spatial abnormalities were a consequence of impaired binaural processing.

Self-reported hearing disability measured via the SSQ in participants with the m.3243A>G variant was not significantly different from controls, but both groups reported considerable difficulty with speech and communication in everyday environments. By comparison, higher average SSQ scores reflective of greater ability/less disability are reported for ‘normal’ hearing younger (19 ± 1 years) and older adults (70 ± 4.1 years): 8.8 ± 0.6 and 7.7 ± 1.2, respectively.^51^ Moreover, our mean SSQ findings are similar to those reported in hearing impaired adults of similar age [54 ± 17 years; SSQ: 6.6 (±2.0)].^52^

A high number of participants (6/10; 60%) who underwent vHIT showed clinically abnormal VOR, with five showing bilateral impairment possibly indicating vestibular dysfunction in this group. Similar findings have been reported in individuals with central disorders such as Cerebellar Ataxia with Neuropathy and Vestibular Areflexia Syndrome, where the vestibular ganglia are impaired leading to abnormally reduced VOR on vHIT testing^53,54^ and in other patient groups with peripheral neuropathy.^28,55^ While abnormal VOR can be the result of vestibular end-organ dysfunction, specifically the semi-circular canals,^56^ this is uncommon and unlikely in patients with other neurological pathologies. Bilateral vestibular deficits often impair individual postural control, locomotion and spatial orientation, with the resulting dizziness and/or falls profoundly impacting patients’ quality of life. This result confirms that vestibular dysfunction is an important clinical manifestation in adults with m.3243A>G mitochondrial disease and hearing impairment, warranting appropriate evaluation and management.

There were two matched control participants who were unable to maintain their balance under the most challenging condition within the mCTSIB. These were the only control participants with abnormal vestibular function (vHIT) (one of which also self-reported mild dizziness). These participants had severe hearing loss with an absent cochlear emission, but normal ABR response.

Participants with the m.3243A>G variant had a significantly higher degree of disability in everyday life imposed by dizziness (DHI sore) when compared with their control counterparts. However, there was no correlation seen between participants self-reported dizziness and VOR gain [as determined by the objective vestibular function test (vHIT)]. This finding of a discordant relationship (between subjective dizziness scales and objective testing) is in line with some studies,^57^ but not others, which have demonstrated a significant negative correlation between subjective DHI scores and VOR gain.^58,59^ The DHI (total and sub-scale scores) has also been reported to be related to patients’ psychological parameters, with anxiety and depression being reported more frequent in individuals with self-reported dizziness.^60^ Compared with healthy controls or norm scores, previous studies report higher scores of depressive and anxiety^61,62^ in patients with m.3243A>G mitochondrial disease.

Recent studies have demonstrated an increased risk of falls when vestibular system impairment is reported alongside impairment in other sensory systems, although the potential influence of hearing loss was not included.^63^ Therefore, it is important to ascertain which systems are involved when patients with mitochondrial disease present with dizziness, impaired balance and/or falls. An expanded vestibular test battery is crucial to determine the extent of vestibular involvement and should incorporate computerized postural control measures to better understand the relationship between sensory systems utilized for postural control. Vestibular rehabilitation has also been shown to be a clinically effective treatment^64-69^; hence, it is important that potential vestibular involvement is assessed as part of a clinical evaluation in mitochondrial disease patients. Minimally invasive diagnosis of vestibular involvement continues to be challenging due to the multi-factorial causes of dizziness, with a recent diagnosis framework unable to reach consensus of six key statements concerning dizziness.^14^ Further research is ongoing to identify minimally invasive diagnostic tools to evaluate the relative importance of different systems in the maintenance of balance in patients with mitochondrial disease, which will enable more effective clinical management.

### Limitations

This study investigated a small sample of patients, limiting its statistical power. Future research in a larger study is required to ensure generalizability. The psychological status of participants (depression and anxiety) was not investigated as a correlate of self-reported symptoms, including dizziness. Patients presenting with dizziness or unsteadiness were not questioned to identify the potential cause/frequency/type of symptoms experienced, which would inform clinical testing and management decisions. Only one clinical vestibular test was used to evaluate the peripheral vestibular system and innervating vestibular nerve.

### Summary and recommendations

In summary, our group of participants with m.3243A>G-related mtDNA disease showed evidence of auditory neuropathy, monaural and binaural processing abnormality and speech perception deficits out of proportion with degree of hearing loss. As such, the findings were consistent, in most individuals, with mixed peripheral/central pathology. Furthermore, each participant reported difficulty with balance and/or dizziness, and most presented with evidence of vestibular impairment.

#### Auditory management

Individuals with m.3243A>G mitochondrial disease demonstrated a specific speech-in-noise impairment. Speech understanding and communication in background noise are improved by increasing the level of the speech signal relative to the competing noise. This can be achieved by remote microphone listening devices, which are designed to improve the signal-to-noise ratio (i.e. the level of the target signal relative to the background noise) at the listener’s ear. Such devices have proven successful in other populations with auditory neural deficits, including Friedreich’s ataxia,^70^ Neurofibromatosis Type 1^71^ and autism spectrum disorder^72^ where conventional amplification has not. The data in our study suggest that auditory evaluation in patients with m.3243A>G mitochondrial disease should include speech perception in noise and an auditory evoked potential assessment where possible, to inform clinical management and improve patient outcomes.

#### Balance and vestibular management

Adults with m.3243A>G mitochondrial disease presenting with hearing impairment and symptoms concerning loss of balance, unsteadiness, falls and dizziness^13^ should undergo evaluation to exclude vestibular involvement. This should include an expanded test battery of vestibular evoked myogenic potentials, vHIT, computerized postural control measures and oculomotor assessment. Where vestibular dysfunction is suspected, patients should be referred for further neuro-otological investigations and have access to appropriate vestibular rehabilitation.^64-69^

## Supplementary Material

fcae361_Supplementary_Data

## Data Availability

Data will be shared upon request with suitably qualified researchers. The corresponding author (G.R.) has full access to the data in the study. Study data will be shared upon request, while maintaining anonymization of participants.
