# Supplementary material for: Auditory and vestibular function in mitochondrial patients harbouring the m.3243A>G variant
Source: Brain Commun. 2024 Oct 14;6(6):fcae361. doi: 10.1093/braincomms/fcae361 (PMC11532826; doi:10.1093/braincomms/fcae361)
Supplement: fcae361_Supplementary_Data [file fcae361_supplementary_data.pdf]

Supplementary Table 1 Demographics and Auditory Brainstem Response findings for individual matched control participants

	Sex	Age (yrs)	Cognition	Cochlear Emissions	Sound detection	Absolute response latencies (msec)			Interpeak-latencies (msec)			Amplitude (μV)	ABR
			ACE-III [a]	DPOAE [b]	AHL (dB)[c]	Wave I	Wave III	Wave V	I–III	III–V	I–V[d]	V/I ratio	Maximum rate[e]
Control 01	F	23	98	Present	17.5	1.46	3.75	5.63	2.29	1.88	4.17	0.82	100
02	M	31	99	Absent	42.5	1.46	3.79	5.29	2.33	1.5	3.83	0.61	100
03	F	34	—	—	51.3	1.5	3.8	5.67	2.3	1.87	4.17	0.52	100
04	F	41	99	Absent	90	1.54	3.74	5.57	2.2	1.83	4.03	0.2	100
05	M	46	95	Absent	70	1.96	4.21	6.21	2.25	2	4.25	0.32	75
06	F	52	99	Absent	55	1.54	3.84	5.67	2.29	1.83	4.13	0.98	100
07	F	50	70	Present	21.3	1.75	3.75	5.5	2	1.42	3.75	0.21	100
08	M	52	87	Absent	52.5	1.59	3.54	5.88	1.96	2.34	4.29	1.31	75
09	F	59	92.6	Absent	82.5	2	4.34	6.34	2.33	2	4.33	0.35	100
10	F	56	95	Present	10	1.63	3.71	5.63	2.08	1.92	4	0.47	100
11	F	59	98	Present	27.5	1.59	3.59	5.5	2	1.92	3.92	0.45	100
12	F	56	96	Absent	52.5	1.34	3.71	5.59	2.38	1.88	4.25	0.33	100

Participant demographics and clinical characteristics and Auditory brainstem response latencies and amplitudes for each of the control participants.

Abbreviations: ABR = Auditory brainstem response; ACE-III = Addenbrooke's cognitive examination III; AHL = audiometrically measured hearing level; DPOAE = distortion product otoacoustic emissions. Response latencies are expressed in milliseconds (ms) and amplitudes are expressed in microvolts (mV)

— not performed

a. ACE-III Total score = sum of item scores. Maximum total score = 100 (18 for attention, 26 for memory, 14 for fluency, 26 for language, 16 for visuospatial); minimum score = 0. Higher scores indicate better cognitive functioning. Score cut-offs for dementia = < 82; mild cognitive impairment= < 88.
b. DPOAE = DPOAE response present: ≥6 dB.
c. AHL = Four frequency average hearing level (0.5 Hz, 1 kHz, 2 kHz, 4 kHz) for the better ear. Normal: ≤15 dBHL; Mild degree hearing loss (21-40 dBHL); Moderate hearing loss (41−70 dB); Severe hearing loss (71−90 dB).
d. ABR = abnormal I-V interpeak latency i.e. conduction time between wave I and V= >4.4 msec.
e. Maximum rate is the highest stimulus presentation rate (Hertz [Hz]) at which an auditory brainstem response could be identified.

Supplementary Table 2 Temporal processing, speech perception and self-reported hearing disability for individuals with m.3243A>G-mitochondrial disease

	Temporal resolution		Monaural speech perception		Binaural speech perception in noise					Self-reported hearing disability			
	AM detection (dB)[a]		CNC Words (%)[b]		LISN-S test, Speech Reception Threshold (dB)[c]					SSQ[d]			
	10 Hz	150 Hz	Quiet	Noise	DV90°	SV90°	DV0°	SV0°	Spatial Adv	Speech	Spatial	Quality	Average
m.3243A>G 01	-15	-18	98.7	58.7	-16	-12.6	-4.5	-1.8	10.8	6.5	4.7	5.5	5.6
02	-25.5	-22.5	94.7	46.7	-1.7	-1	0.4	2.8	3.8	4.2	7.3	9.2	6.9
03	-15	-10.4	97.3	36.7	-1.4	-1.2	2.5	3.1	4.4	—	—	—	—
04	—	—	16	0	0	1.1	1.5	1.2	0.1	2.9	4.3	4.5	3.9
05	-12	-6	78.7	10.7	6.1	7.5	3.7	6.3	-1.2	5.1	8.4	7.5	7.0
06	-10.5	-6	86.7	20	4.4	3.2	0.8	3.9	0.6	6.4	6.4	7.9	6.9
07	-18	-16.5	87.3	54.7	-16.8	-16.9	-5.7	-1.4	15.5	6.0	6.6	7.3	6.6
08	-9	-7.5	80	24	1.9	2.8	1.9	3.2	0.5	1.0	3.9	6.1	3.7
09	-22.6	-4.4	12	0	4	4.3	2.3	3.8	-0.5	3.1	1.9	2.1	2.4
10	-12	-15	97.3	40	-10.6	-8.3	-1	0.2	8.5	7.6	8.8	9.3	8.6
Control 01	-20.2	-18.4	84	56.7	-17	-13.9	-1.4	-0.9	13.1	4.4	6.5	5.6	5.5
02	-22.4	-19.4	82.7	36	-12.6	-6.5	-5.6	-1.1	5.4	5.9	4.9	6.4	5.7
03	-22.4	-19.5	80	40	-12.5	-10.8	-4.8	-2.8	8	—	—	—	—
04	—	—	70.7	34	5.9	5.9	4.5	10.7	4.7	5.1	4.1	7.2	5.5
05	-18	-13.4	86.67	37.3	2.4	2.4	4	5.2	2.9	6.9	6.8	6.1	6.6

06	-18	-15	57.3	40	-7.7	-5.9	-1	0.5	6.5	8.1	5.9	8.3	7.5
07	-18	-19.5	93.3	59.3	-12.5	-12.8	-4.1	0.6	13.4	5.4	7.7	6.8	6.7
08	-16.4	-10.4	74.7	20	-1.82	-1.5	-0.1	2.1	3.54	7.1	2.1	2.5	3.9
09	-20.2	-16.4	68	34.67	4.2	5.8	4.5	7.6	1.8	4.5	4.2	5.9	4.9
10	-19.5	-18.4	98.7	62	-14.6	-15.9	-9.2	-1.2	14.71	9.1	9.5	9.6	9.4

Temporal processing, speech perception and self-reported hearing disability for participants with m.3243A>G-mitochondrial disease and matched control participants.

Abbreviations = AM = amplitude-modulation; CNC Words = Consonant Nucleus Consonant; DV90°, different voices (target sentence and background) separated by 90°; DV0°, different voices, same direction; LISN-S = Listening in Spatialised Noise-Sentences test; Spatial adv = Spatial Advantage, improvement in Speech Reception Threshold when the target speech and noise are spatially separated; SSQ = The Speech, Spatial and Qualities of Hearing Scale; SV90°, same voice separated by 90°; SV0°, same voice, same direction.

— not performed

a. AM depth threshold = smallest detectable modulation depth (dB) in a burst of white noise.
b. CNC Words Test = phoneme score (% correct). Quiet: Abnormal = < 89.5 %; Noise (0 dB SNR): Abnormal = < 36.2 %.
c. Speech Reception Threshold = the signal-to-noise ratio required to identify 50% of the words in a target sentence.
d. SSQ = Maximum average score = 10; minimum score= 0. Higher scores reflect greater ability (less disability).

Supplementary Table 3 Vestibular function, balance and self-reported dizziness for individual matched control participants

		mCTSIB[b]		VOR[c]				
	AHL (dB)[a]	Seconds	Comments	Left	Right	Test ear	vHIT Comments	DHI Total[d]
Control 01	17.5 (Mild)	120	Normal	0.86	1	1.0	Normal HSCC (bilateral)	0
02	42.5 (Moderate)	120	Normal	0.9	1.01	1.01	Normal HSCC (bilateral)	12
03	51.3 (Moderate)	—	—	—	—	—	—	—
04	90 (Severe)	106	Abnormal Condition: 4	0.56	0.4	0.56	HSCC dysfunction (bilateral)	0
05	70 (Moderate)	120	Normal	0.87	0.96	0.87	Normal HSCC (bilateral)	2
06	55 (Moderate)	120	Normal	0.87	0.92	0.87	Normal HSCC (bilateral)	16 (Mild)
07	21.3 (Mild)	120	Normal	1.1	0.89	1.1	Normal HSCC (bilateral)	18 (Mild)
08	52.5 (Moderate)	120	Normal	0.82	0.98	1	Normal HSCC (bilateral)	8
09	82.5 (Severe)	103	Abnormal Condition: 4	0.52	0.74	0.74	HSCC dysfunction (left)	34 (Mild)
10	10 (Normal)	120	Normal	0.93	1.04	0.93	Normal HSCC (bilateral)	0
11	27.5 (Mild)	120	Normal	0.92	1.08	0.92	Normal HSCC (bilateral)	0
12	52.5 (Moderate)	120	Normal	1.04	1.01	1.01	Normal HSCC (bilateral)	10

Sound detection, vestibular function, balance and self-reported dizziness for each of the control participants.

Abbreviations: AHL = audiometrically measured hearing level; DHI, Dizziness Handicap Inventory; HSCC, horizontal semicircular canal; mCTSIB = modified Clinical Test of Sensory Interaction in Balance; vHIT, video head impulse test; VOR, vestibulo-ocular reflex.

— not performed

a. AHL = Four frequency average hearing level (0.5 Hz, 1 kHz, 2 kHz, 4 kHz) for the better ear. Normal: ≤15 dBHL; Mild degree hearing loss (21-40 dBHL); Moderate hearing loss (41−70 dB); Severe hearing loss (71−90 dB).
b. mCTSIB: Time maintaining balance across 4 conditions, with 30 seconds the maximum duration for each condition. Abnormal = if there was more than one loss of balance across all trials. Condition One = Eyes Open, Firm Surface; Condition Two = Eyes Closed, Firm Surface; Condition Three = Eyes Open, Foam Surface; Condition Four= Eyes Closed, Foam Surface.
c. VOR Gain Abnormal= < 0.80.
d. DHI Total score= sum of item scores. Maximum total score= 100 (28 for physical, 36 for emotional, 36 for functional) minimum score = 0. Higher scores indicate greater perceived disability. Handicap cut-off scores, Mild= 16-34; Moderate= 36-52; Severe= ≥ 54.
